# Developing an immunotherapy strategy for the effective treatment of patients with squamous cell carcinoma of the head and neck

**DOI:** 10.1186/2051-1426-1-S1-P262

**Published:** 2013-11-07

**Authors:** Tarsem Moudgil, R Bryan Bell, Rom Leidner, Rieneke Van de Ven, Zipei Feng, Michael Affentoulis, Christopher Paustian, Christopher Dubay, Traci L Hilton, Walter J Urba, Hong-Ming Hu, Carlo B Bifulco, Bernard A Fox

**Affiliations:** 1 Robert W. Franz Cancer Research Center, Earle A. Chiles Research Institute, Portland, OR, USA; 2 Oral, Head and Neck Cancer Program and Clinic, Providence Cancer Center, Portland, OR, USA; 3 Molecular Microbiology and Immunology, OHSU, Portland, OR, USA; 4 UbiVac, Portland, OR, USA; 5 Department of Pathology, Providence Cancer Center, Portland, OR, USA

## 

Squamous cell carcinoma of the head and neck (HNSCC) arises in the oral cavity, oropharynx, larynx or hypopharynx, and is the 6th leading cause of cancer by incidence worldwide. Approximately 600,000 cases arise each year, and only 40-50% of these patients will survive for 5 years.7 HNSCC can express PD-L1 and suppressive factors that interfere with the differentiation and activation of dendritic cells and effector function of T cells. Similar to observations in other malignancies, CD8 effector T cell infiltration or transcriptional signature consistent with an ongoing T cell response is associated with prolonged survival. This suggests that HNSCC lacking a T cell infiltrate may benefit from immune interventions that induce and/or augment a tumor-specific immune response. To develop a strategy to address this problem we have created a HNSCC tumor bank that is cryopreserving enzymatically isolated viable cells from resected tumors (39 specimens). We are also attempting to develop primary cell lines (4 lines established) and are isolating tumor-infiltrating lymphocytes from these specimens. We are looking to compare immunohistochemical, flowcytometric and functional analyses of these specimens. Consistent with previous studies, results document that isolated TIL secrete IFN-γ when stimulated with their autologous tumor cells (n=2). Since a central aim of these studies is to develop a strategy to create therapeutic immunity in patients lacking T cell infiltrates, we plan to investigate which antigens are recognized and whether, antigens present in a novel DRibble vaccine, that expresses antigens common to HNSCC, are recognized by these TIL. This may provide a strategy to monitor anti-tumor immunity in these patients and possibly provide an antigen source for combination immunotherapy of patients with HNSCC.

